# Methamphetamine Users Show No Behavioral Deficits in Response Selection After Protracted Abstinence

**DOI:** 10.3389/fpsyt.2019.00823

**Published:** 2019-11-19

**Authors:** Wiebke Bensmann, Julia Ernst, Marion Rädle, Antje Opitz, Christian Beste, Ann-Kathrin Stock

**Affiliations:** Cognitive Neurophysiology, Department of Child and Adolescent Psychiatry, Faculty of Medicine, TU Dresden, Dresden, Germany

**Keywords:** dopamine, error processing, flanker effect, Gratton effect, methamphetamine abstinence, response selection

## Abstract

**Introduction:** Chronic recreational methamphetamine use causes dopaminergic neurotoxicity, which has been linked to impairments in executive functioning. Within this functional domain, response selection and the resolution of associated conflicts have repeatedly been demonstrated to be strongly modulated by dopamine. Yet, it has never been investigated whether chronic methamphetamine use leads to general impairments in response selection (i.e., irrespective of consumption-associated behavior) after substance use is discontinued.

**Materials and Methods:** We tested n = 24 abstinent methamphetamine users (on average 2.7 years of abstinence) and n = 24 individually matched controls in a cross-sectional design with a flanker task.

**Results:** Compared to healthy controls, former methamphetamine consumers had significantly slower reaction times, but did not show differences in the size of the flanker or Gratton effect, or post-error slowing. Complementary Bayesian analyses further substantiated this lack of effects despite prior consumption for an average of 7.2 years.

**Discussion:** The ability to select a correct response from a subset of conflicting alternatives, as well as the selective attention required for this seem to be largely preserved in case of prolonged abstinence. Likewise, the ability to take previous contextual information into account during response selection and to process errors seem to be largely preserved as well. Complementing previously published finding of worse inhibition/interference control in abstinent consumers, our results suggest that not all executive domains are (equally) impaired by methamphetamine, possibly because different cognitive processes require different levels of dopamine activity.

## Introduction

Amphetamines are the second most commonly used illicit drugs worldwide and out of all amphetamines, methamphetamine is considered to represent an especially large threat to global health (1). Low to moderate oral doses of methamphetamines actually improve cognitive functioning and lead to various mental and physical effects including a positive mood, euphoria, and reduced fatigue (2). In case of repeated consumption, consumers experience an attenuationof these pleasant (acute) effects due to a development of tolerance, and rapidly become dependent (3–7). Repeated administration of large doses, as usually observed in substance use disorder, are associated with multiple deleterious medical consequences including psychosis, cardiovascular problems, nutritional deficiencies, sleep deprivation, and decreased cognitive functioning (e.g. 2, 8, 9–11).

These effects have been associated with acute increases in monoaminergic signaling and neurotoxic effects of the drug on the dopamine system (12–14). There is strong evidence that methamphetamine increases the release of monoamines *via* uptake transporters (2, 15), which leads to enhanced presynaptic release and heightened postsynaptic receptor binding (16, 17, 14). Prolonged use however results in the opposite, i.e., substantial reductions in presynaptic monoamine transporters and postsynaptic monoamine receptors, which effectively downregulate the dopaminergic system (18–20). Importantly, clinical markers of this pathology (like reduced DAT binding) have been shown to likely take more than a year to recover (21, 20), which suggests cognitive deficits that are associated with this dopaminergic dysfunction should also take at least a year, if not more, to recover.

In line with this, previous studies have suggested that dopamine-associated cognitive deficits may extend well into abstinence. In early stages of abstinence, deficits are comparable to those seen in currently abusing individuals across different domains of executive functioning. This includes cognitive flexibility, working memory and, perhaps to a greater extent, inhibitory control, as shown by deficits in the Wisconsin Card Sorting Test 22, 23), Digit Span Test (24, 25), and Stroop Task (26–28). These functions are pivotal for controlling substance intake (29) as well as for driving behavioral changes in the face of negative consequences (30). Yet still, it has remained rather unclear whether abstinent methamphetamine users also show behavioral differences in response selection that extend beyond consumption-associated behavior. This question is of great functional relevance, as the ability to select a correct response among several competing response alternatives and to resolve conflicts that arise between such options is a key prerequisite to goal-directed behavior (31). It has previously been demonstrated that the mental representation of behavioral goals/mental task sets depends on the input/output function of prefrontal cells, which is effectively modulated by dopamine (32) and plays an important functional role for response selection (33). As dopamine improves gain control mechanisms by amplifying the brain’s ability to efficiently process input signals and reduce neuronal noise (34, 35), the dopamine deficiency reported in former methamphetamine users may render them unable to efficiently select responses and resolve response conflicts, or selectively attend to task-relevant information. Yet, research on potential response selection deficits in former methamphetamine users is still scarce. It has however been shown that methamphetamine seems to impair attentional processing (36, 26), and that cocaine users show functional deficits in error processing (37) which qualitatively resemble those of Parkinson’s patients (38) and Huntington’s patients (39). Furthermore, there is evidence that functional changes in dopaminergic signaling modulate response selection in different versions of the flanker tasks (40–42).

For this reason, we used a version of the Eriksen flanker task (43–45) to assess potential differences in response selection, attention, and error processing between former abstinent methamphetamine users and matched drug-naïve controls. The paradigm allows to investigate response conflicts and attention with the help of the flanker effect, as well as “carry-over” effects of previous contextual information with the help of the Gratton effect. While the flanker effect is characterized by better performance in trials with congruent flanker stimuli (as compared to incongruent trials, see 45), the Gratton effect is characterized by an interference effect of conflicts in the previous (n-1) trial on the current trial (n): Typically, the flanker congruency effect in the current trial (n) is smaller in case of an incongruent previous (n–1) trial (as compared to a congruent previous/n-1 trial) (46, 47). Additionally, the task allows to investigate error processing with the help of the post-error slowing (PES) measure, which has also been shown to be modulated by dopamine (48–52). Increases in dopamine signaling seem to improve response selection/decrease flanker effects (40, 41), while decreases in dopaminergic signaling likely impair response selection. In line with this, it has been suggested that patients with Parkinson’s disease, who have a strong dopamine deficit, seem to show larger flanker effects under speed stress (53). Moreover, the Gratton effect was demonstrated to be modulated by dopamine, as shown by eye blinks as putative markers for dopamine (54). In line with this, it has been reported that patients with Parkinson’s disease do not show the Gratton effect (55). We hence hypothesized that the supposedly dopamine-deficient abstinent methamphetamine users might not only show general control deficits but might also show larger flanker and Gratton effects, as compared to drug-naïve controls. Potential differences in post-error slowing were analyzed in an exploratory fashion.

Last but not least, it should be noted that several studies have shown that the downregulation of the dopamine system improves with protracted abstinence from methamphetamine (20, 56). While these studies have demonstrated remarkable improvement/normalization from <6 months to 12 to 17 months of abstinence, it has been reported that residual deficits could still be observed after 12 to 17 months of abstinence (20, 56). We hence decided to not limit our inclusion criteria to a certain abstinence duration. In this context, it should be noted that even after prolonged abstinence of more than 18 months on average, former methamphetamine consumers may still show deficits in inhibitory control (as reflected by a Stroop task) and in beneficial disengagement of working memory-associated control functions (assessed in a meta-control paradigm) (57).

In short, the main objective of the current study was to investigate whether former methamphetamine users show deficits in response selection, selective attention, or error processing during prolonged abstinence. For this purpose, we applied the Eriksen flanker task to a self-reporting sample of abstinent methamphetamine users and drug-naïve controls, who had been individually matched for sex, age, and education. We hypothesized to find larger flanker and Gratton effects in former methamphetamine users due to the dopaminergic toxicity of the drug.

## Methods

### Sample

A group of n = 32 adult former/abstinent methamphetamine consumers (mean age 29.5; SD 5.04; range 20 to 38 years; 11 females) took part in this study. There were several inclusion criteria: All participants should have consumed methamphetamine at least three times a week for at least six consecutive months of their life and have experienced both craving and withdrawal symptoms during this time. Participants should consider themselves as former drug addicts and methamphetamine should be the main substance of addiction. All participants should be abstinent from methamphetamine, amphetamine, or other illicit drugs for at least two weeks prior to their first appointment, and should have started abstinence on their own or with the help of a medical care program (i.e. not for the purpose of this study). Participants should report no psychological or pharmacological treatment for coping with addiction or withdrawal at the time of data collection. However, past psychological treatment/counseling was not assessed. An experienced psychologist confirmed the ICD10 F15.2 diagnosis of methamphetamine dependence with current abstinence on the first study appointment. Moreover, participants had to be free from (diagnosed) psychiatric disorders or neurological diseases before they started consuming methamphetamine. Inclusion criteria concerning current mental health or additionally consumed substances during methamphetamine use were less strict, as long as methamphetamine was clearly the main substance of abuse and an experienced psychologist expected only minor or no task performance impairments due to psychiatric symptoms. While this certainly increased the variance within the sample, it also provides a more realistic picture of cognitive effects in former consumers.

We further recruited n = 32 healthy adults as a drug-naïve control group (mean age 29.3; SD 5.66; range 18 to 39 years; 11 females), which had been individually matched to an assigned former consumer with respect to sex, age (max ± 2.5 years) and education (a maximal difference of one educational or vocational degree was tolerated). Control participants reported to have no psychiatric, neurologic, or chronic diseases, no lifetime experience with any kind of illicit substance (e.g. methamphetamine, amphetamine, speed, MDMA, methylphenidate, cocaine etc.) and never have received a diagnosis of drug addiction or substance use disorder.

All participants had normal or corrected-to-normal vision. The study would only be conducted when abstinence from illicit drugs was confirmed by negative urine drug screenings using “nal von minden Drug-Screen” tests (nal von minden GmbH, Regensburg, Germany) for amphetamines, methamphetamine, morphine, and THC. Moreover, the participants had to present with a BAC of 0.00 ‰, as assessed with the help of the “Alcotest 3000” breath analyzer following the instructions of the manufacturer (Drägerwerk, Lübeck, Germany), and showed no obvious signs of withdrawal at all study appointments. Any participants who failed to present entirely sober upon any given study appointment would have been excluded from study participation.

After data collection, we had to exclude n = 8 former methamphetamine consumers for the following reasons: One participant reported a traumatic brain injury during childhood, one complained about impaired vision, two provided incoherent information about their addiction and consumption history and/or did not sufficiently meet the diagnostic criteria for (former) dependence, so that we could not assume previous addiction with sufficient certainty. Four more participants had to be excluded because they failed to perform the task above chance level and we could not determine the origin of these issues with certainty when analyzing the data (i.e., retrospectively determine whether these participants failed to understand the task instructions, or whether they failed to comply with them for various possible reasons). As methamphetamine users and controls had been matched individually, we also excluded all drug-naïve controls who had been matched to an excluded consumer. Each participant gave written informed consent and was reimbursed with 50€ for taking part in the study. The study was conducted in accordance with the Declaration of Helsinki and approved by the ethics commission of the Medical Faculty of the TU Dresden.

### First Study Appointment: Interview, Questionnaires, and Neuropsychological Tests

During the first of two study appointments, we assessed sociodemographic data, (illicit) substance consumption, potential comorbid psychiatric disorders, and executive functioning with the help of several questionnaires, structured interviews, and paper-pencil tests.

First, an experienced neuropsychologist assessed whether several psychiatric disorders such as depression or psychotic episodes were likely to be present in any of the participants with the help of the M.I.N.I. International Neuropsychiatric Interview (58). Afterwards, participants were asked to fill in Beck’s depression inventory (BDI; 59) to assess potential depression symptoms which might interfere with cognitive performance. Subsequently, the subjects had to perform neuropsychological tests to assess overall cognitive functioning: A verbal version of the Stroop task (60) was conducted to measure inhibition and interference control. The trail-making test (TMT) (61) was used to measure cognitive flexibility and task set switching. To assess short-term memory and working memory in the verbal and spatial domain, the digit span test from the WAIS-IV test battery (62; 63) and the Corsi block span test (64; 65) were conducted. To collect sociodemographic and health-related data, participants had to fill in customized questionnaires. Furthermore, the Alcohol, Smoking, and Substance Involvement Screening Test (ASSIST) (WHO ASSIST Working 66) was conducted with each participant to assess lifetime prevalence for common drugs of abuse and their use within the last three months preceding the appointment. To check for any inconsistencies or contradictions concerning addiction and abstinence, experienced psychologists discussed the individual addiction history with each of the self-reported abstinent addicts in the methamphetamine group.

### Second Study Appointment: Experimental Paradigm

**Figure 1:**
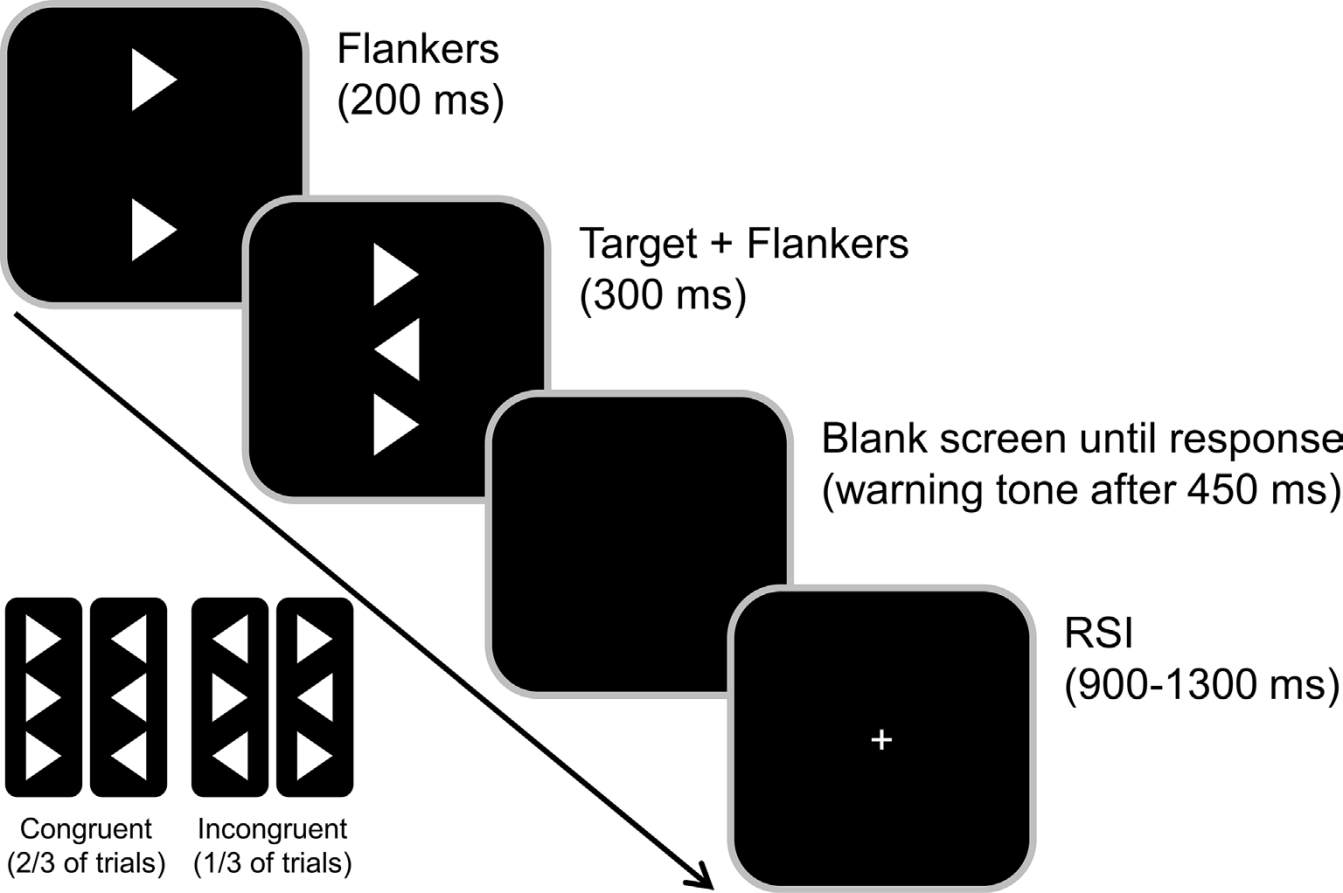
Illustration of the flanker task. The congruent and incongruent flanker arrowhead appeared 200 ms before the target onset (middle arrowhead). Participants were asked to indicate the target direction by pressing the Ctrl button on the respective side of a standard keyboard. The response-stimulus interval (RSI) randomly varied between 900 and 1300 ms.

A standard flanker task was used (43, 44) to investigate response conflicts and carryover effects of previous contextual information (see **Figure 1**). Participants were seated at a 57 cm distance from a 17-inch CRT monitor and were asked to respond using a QWERTZ keyboard. We used Presentation software (Version 17.1 by Neurobehavioral Systems, Inc.) to present the stimuli and record behavioral responses. Before the start of the paradigm, subjects practiced the task until both the participant and the experimenter were confident that the task could be performed as instructed. Participants were asked to rest their index fingers on the response buttons (right and left Ctrl buttons) and react to the target as quickly and accurately as possible.

The target stimulus was a white arrowhead that was displayed in the center of the screen on a black background and either pointed to the left or the right. The target was flanked by two vertically aligned arrowheads, that either pointed in the same direction as the target (congruent) or in the opposite direction (incongruent). These flanker stimuli preceded the target by 200 ms so that the stimulus-onset asynchrony (SOA) was 200 ms. The target and flanker stimuli were then presented for 300 ms and switched off simultaneously. The response-stimulus interval between the first response and the onset of the following trial was jittered between 900 and 1,300 ms. To further increase task difficulty and given that time pressure might be required to see behavioral effects of dopamine deficiency (53), time pressure was administered by asking the participants to respond within 450 ms. In trials where the reaction time exceeded this deadline, an auditory warning stimulus (1000 Hz, 60 dB SPL) was given after this time interval. The subjects had to perform four blocks of 120 trials each. Of these 480 trials, 67% were congruent, and 33% were incongruent trials.

### Statistics

Separate mixed effects ANOVAs were performed to analyze the behavioral data. All analyses used current trial (congruent vs. incongruent) as within-subject factor and consumption group (meth vs. control) as between-subject factor. Accuracy and hit RT analyses also used previous (n-1) trial (congruent vs. incongruent) as within-subject factor. PES did not use previous trial, as we did not have enough incorrect responses to reliably analyze this factor in that measure. To investigate the effects of abstinence duration, we additionally ran separate analyses in the meth consumption group only (i.e., when excluding all controls) using the between-subject factor abstinence subgroup (short vs. long abstinence). The degrees of freedom were adjusted using Greenhouse-Geisser correction, and results were Bonferroni-corrected, whenever necessary.

Potential group differences in scores of neuropsychological tests and questionnaires were analyzed with the help of independent samples *t*-tests whenever the scores were normally distributed, as assessed with KS tests. If this criterion was not met, Mann-Whitney *U* tests were used instead. Please note that we did not apply Bonferroni corrections because these tests were only exploratory and not used to answer the main research question of this study.

For all descriptive statistics, the mean and the standard error of the mean (SEM) are given as a measure of variability.

## Results

### Sample Description of Former Methamphetamine Consumers

The *n* = 24 included abstinent methamphetamine consumers started consuming methamphetamine at the mean age of 18.4 years (± 3.9; range 13 to 30) and consumed it for 86.2 months (± 47.8; range 12 to 216), i.e. approximately seven years on average. Out of those 86.2 months, they recreationally used methamphetamine (as defined by irregular use, a subjective lack of withdrawal, craving, or negative social or occupational consequences, as well as the absence of drug-related crimes) for an average of 25.1 months (± 18.3; range 0 to 60) and reported having been subjectively addicted (as defined by regular use, the subjective presence of withdrawal and/or craving and negative social or occupational consequences) for an average of 59.0 months (± 36.1; range, 6 to 144). It should, however, be noted that all of this information was assessed retrospectively and may therefore not always accurately depict past events. The mean abstinence duration was 31.9 months (± 30.7; range 1.5 to 120). We also performed a median split of the methamphetamine group, thus forming a short and long abstinence subgroup to investigate the effects of abstinence duration. As each subgroup, however, only contained only n = 12 subjects and does therefore not have enough statistical power to allow for strong conclusions (67), we only provide these results in the **Supplementary Material**. Of note, this study’s sample has previously been used to investigate meta-control and disengagement of control whenever automaticity would be most beneficial for behavioral performance in a working memory-modulated context (57). In that previous study, the data obtained from the assessed questionnaires and neuropsychological paper-pencil tests has already been published (57). But while there is a great overlap between the two samples, they still differed with respect to which subjects were excluded from the sample based on task performance. As a consequence, the results obtained in this sample are similar, but not identical to the previously published data. Hence, all of the information assessed in these tests can also be found in the **Supplemental Material**.

### Behavioral Results

**Figure 2:**
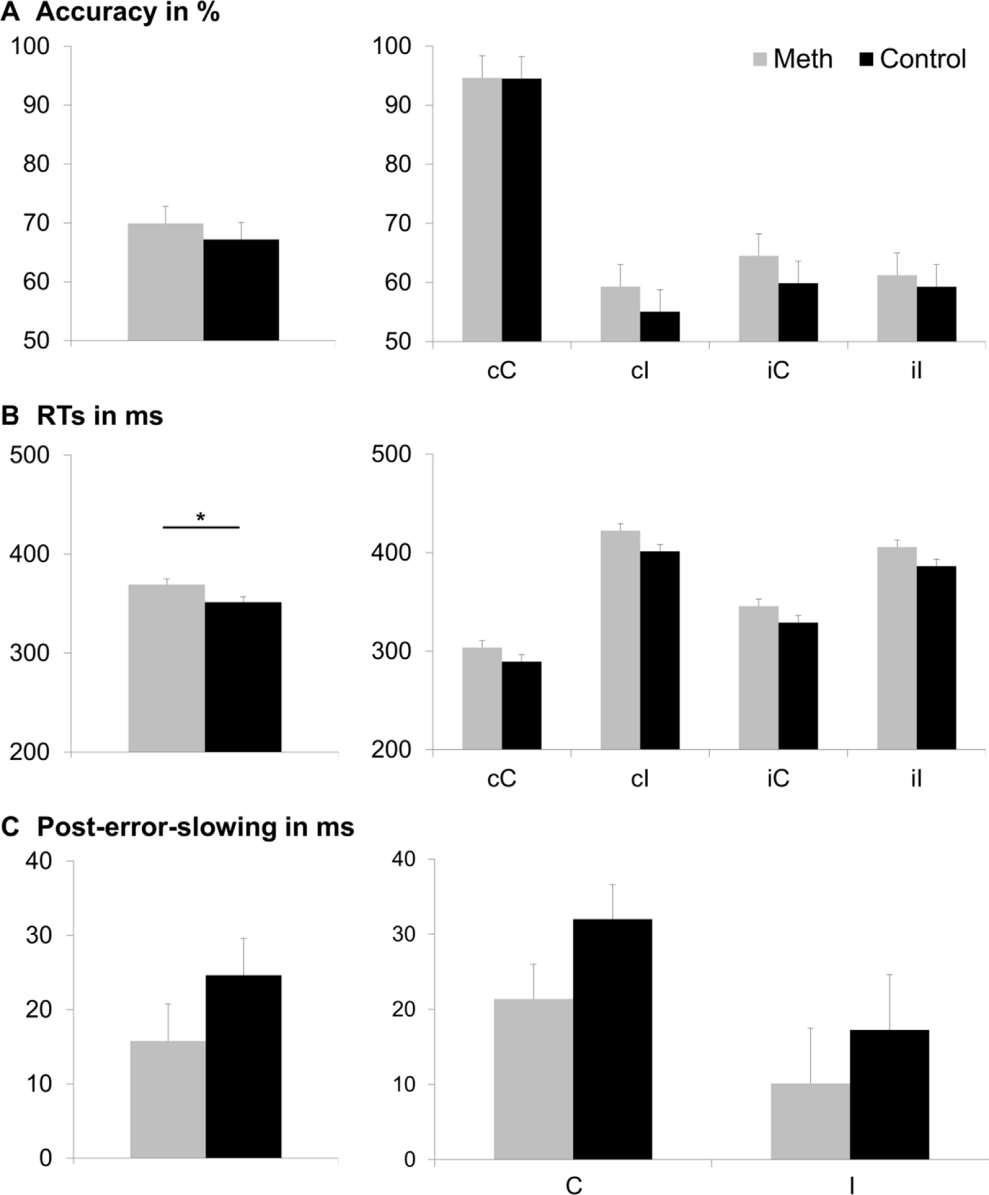
Behavioral results. Part **(A)** displays accuracy (percentage of correct responses), part **(B)** displays hit reaction times (RTs in ms), and part **(C)** displays post-error-slowing (PES in ms). The main effects of consumption group (Meth vs. Control) are displayed in the left column. The group difference was only significant for hit RTs (p ≤ .05; denoted with an asterisk), but not for accuracy or PES. For accuracy and hit RTs, each combination of previous trial (first letter: c = congruent; i = incongruent) and current trial (second letter: C = congruent; I = incongruent) is depicted separately in the middle and right columns. For PES, we only assessed the effect of the current trial (C = congruent; I = incongruent). Consumption group (Meth vs. Control), did not significantly interact with any of these factors. Error bars show the standard error of the mean (SEM) as a measure of variability.

The behavioral data of the flanker task is illustrated in **Figure 2**. The analysis of accuracy (percentage of hits) revealed a main effect of previous trial [F(1,46) = 146.70, p < .001, =761]: Participants responded less accurately when the previous trial was incongruent (61.22% ± 2.58) than when the previous trial was congruent (75.87% ± 1.53). Moreover, there was a significant main effect of current trial [F(1,46) = 159.65, p < .001, = 776], with a higher accuracy in congruent (78.37% ± 1.43) than in incongruent current trials (58.71% ± 2.72). Additionally, there was an interaction of previous trial and current trial [F(1,46) = 215.34, p < .001, = 824]. Post-hoc t-tests revealed that the flanker effect (i.e. congruent minus incongruent current trial) was significantly smaller when the previous trial had been incongruent (1.94% ± 1.38), as compared to when the previous trial had been congruent (37.38% ± 2.40) [t(47) = −14.53; p < .001]. All other main effects and interactions, including those of consumption group, were not significant (all F ≤ 1.971; p ≥.167).

With respect to response times (RTs), the repeated measures ANOVA revealed a main effect of consumption group [F(1,46) = 5.11, p = .028, = 100], indicating that RTs were longer in the meth group (369 ms ± 5) than in the control group (351 ms ± 5). Given the recent debate about the need for larger sample sizes in psychology (67), we additionally ran post-hoc power analyses using G-power software (www.gpower.hhu.de/) (68). These analyses informed us that the sample yielded a power of 77% (at α = 5% when entering the obtained effect size [f = 0.33] and item inter-correlation [r = 0.606]). There was also a main effect of previous trial [F(1,46) = 43.33, p < .001, = 485], showing faster RTs when the previous trial was congruent (354 ms ± 4), than when the previous trial was incongruent (366 ms ± 4). In addition, there was a main effect of current trial [F(1,46) = 387.75, p < .001, = 894], revealing faster RTs when the current trial was congruent (316 ms ± 4) than when the current trial was incongruent (403 ms ± 4). Furthermore, an interaction of previous trial and current trial was obtained [F(1,46) = 118.88, p < .001, = 721]. Post-hoc t-tests showed a significantly larger flanker effect (i.e. incongruent minus congruent current trial) in case of a congruent previous trial (115 ms ± 4) than in case of an incongruent previous trial (58 ms ± 5) [t(47) = −11.01; p < .001]. All other main effects and interactions of the RT analyses, including consumption group, were not significant (all F ≤ 4.021; p ≥ .051).

For the PES, there was a main effect of current trial [F(1,46) = 6.56, p < .014, = 125], with a higher PES in congruent (26.6 ms ± 3.2) than in incongruent trials (13.6 ms ± 5.2). All other main effects and interactions, including those of consumption group, were not significant (all F ≤ 1.57; p ≥ .216).

Non-significant results obtained with regular null hypothesis statistical testing are hard to interpret and should therefore be treated with caution. To substantiate the assumption that the consumption groups did indeed not differ in the assessed task measures, we conducted additional Bayesian analyses as suggested by Wagenmakers (69) using the template by Masson (70). These analyses require a transformation of sum-of-squares values generated by the standard analysis of variance. This approach generates a graded level of evidence indicating which model (e.g., effect absent versus effect present) is more strongly supported by the data. This analysis yields the value of pBIC(H_0_|D), which is the probability of the null hypothesis being true, given the obtained data. Values below .5 are in favor of the alternative hypothesis (i.e., indicate that the alternative hypothesis is more likely to be true than the null hypothesis). Values between .5 and .75 are interpreted as weak evidence, values between .75 and 95 are interpreted as positive evidence, values between .95 and .99 are interpreted as strong evidence, and values above .99 are interpreted as very strong evidence in favor of the null hypothesis (71). The results obtained in our Bayesian analysis of consumption group effects are summarized in **Table 1**.

**Table 1:** Bayesian analyses for all effects involving the consumption group factor.

	*Accuracy in %*	*RTs in ms*	*PES in ms*
*Main effect consumption group*	pBIC(H_0_|D) = .846	pBIC(H_0_|D) = .355(*)	pBIC(H_0_|D) = .756
*Current trial x consumption group*	pBIC(H_0_|D) = .917	pBIC(H_0_|D) = .885	pBIC(H_0_|D) = .867
*Previous trial x consumption group*	pBIC(H_0_|D) = .897	pBIC(H_0_|D) = .922	
*Previous trial x current trial x consumption group*	pBIC(H_0_|D) = .369	pBIC(H_0_|D) = .907	

*The main effect of consumption group was significant in the ANOVA.

pBIC(H_0_|D) is the probability of the null hypothesis being true, given the obtained data. Please note that no data on the effects of previous trial are reported for post-error slowing (PES), as we did not have enough incorrect responses to reliably analyze this factor.

Most of these results provide greater evidence, and most often even positive evidence for the null hypothesis (i.e. no differences between consumption groups) and thus the rejection of the alternative hypotheses (i.e., differences between consumption groups) for all non-significant main and almost all non-significant interaction effects of the consumption group factor.

## Discussion

Chronic recreational methamphetamine use has repeatedly been suggested to cause impairments in executive functioning *via* dopaminergic neurotoxicity, which may (to a certain degree) prevail even over longer periods of abstinence (2, 15). Since dopamine plays a very important role in response selection (72), it may be assumed that these processes show deficits in methamphetamine users even after the initiation of abstinence. We used a version of the Eriksen flanker task (43–45) to assess potential differences in response selection between abstinent former methamphetamine users and drug-naïve controls, who had been individually matched for sex, age, and education. We had hypothesized that compared to drug-naïve controls, abstinent methamphetamine users might show larger flanker and Gratton effects. This could possibly be due to the supposed dopamine deficiency.

In the current study, we were able to reproduce the flanker effect as well as the Gratton effect (40, 41, 43, 45). Moreover, abstinent methamphetamine users had significantly slower reaction times than healthy controls, indicating a general decrease in performance, as compared to drug-naïve controls. Add-on Bayesian analyses of this effect provided weak evidence for the alternative hypothesis being true, given the data (pBIC(H_1_|D = .645). However, we did not find any other significant behavioral differences between abstinent methamphetamine users and controls, or any significant interaction of the consumption group factor with any of the experimental manipulations/conditions. Further substantiating this lack of effects, post-hoc Bayesian add-on analyses confirmed that there was stronger (and most often, positive) evidence of the null hypothesis (H_0_), thus indicating that there is likely no behavioral performance difference between abstinent methamphetamine users and controls in the domain of response selection. The only exception from this was the interaction between Gratton effect, flanker effect, and consumption group, where Bayesian analysis was slightly more in favor of the alternative hypothesis, but did not provide strong support for the alternative, either (pBIC(H_1_|D = .631). Also, this interaction did not reach significance.

These results obtained in the current study are hence not in line with previous studies demonstrating significantly reduced executive functioning in former methamphetamine users (e.g. 73): Using the Wisconsin Card Sorting Test (22, 23), Digit Span Test (24, 25), and Stroop Task (26–28), several studies suggested executive control deficits in methamphetamine users who have been abstinent for more than one month. Specifically, these studies demonstrated detrimental effects of methamphetamine on cognitive flexibility, working memory and, perhaps to a greater extent, inhibitory control. Yet still, our findings are not entirely at odds with these findings, as we were able to reproduce the repeatedly reported worsening of inhibitory control in the Stroop Task. Specifically, we found abstinent methamphetamine users to take significantly longer than controls to complete the conflict condition (Please find additional information on these findings in Stock, (74) and in the supplement) (compare 75–78). It should however be noted that this aspect of our findings had already been previously published elsewhere (57).

Nonetheless, our findings on response selection support a growing body of literature suggesting that former methamphetamine (ab)use does not necessarily influence all cognitive domains in the same way, or to the same extent. Deficits found in inhibitory control and cognitive flexibility can therefore not necessarily be generalized to response selection and error processing. One possible explanation is related to the underlying dopaminergic mechanisms: Dopamine seems to modulate executive functions in the fashion of an inverted U-shaped curve (79, 80). There seems to be an optimal level of dopamine, where input signal processing and neural noise reduction are most efficient. As a consequence, both too low and too high concentrations of dopamine may lead to a decline in gain control (34, 35, 81) and finally in behavioral performance (e.g. 79, 80). In this context, it could be demonstrated that the dopamine level which is optimal for performance, depends on baseline task performance as well as the difficulty of a given task (82–84). As a consequence, the amount of gain control required for a task might also differ. This makes it reasonable to assume that the optimal dopamine level depends on the cognitive domain tested in a given task. In other words, the optimal level of dopamine needed for optimal Flanker task performance might be lower than for the Stroop Task. If this was the case, it could explain why the behavioral performance of abstinent methamphetamine consumers differed from drug-naïve controls in the Stroop task, but not in the Flanker task. While the Flanker Task and Stroop Task are both regarded as measure of response inhibition, they are nonetheless functionally different: The Stroop Task assesses interference control *via* two interfering stimulus dimensions (font color and written word) of the same target stimulus, which simultaneously compete for cognitive resources and thereby induce a conflict (31). The flanker task, however, requires to shield task-relevant information provided by a target stimulus from distracting bottom-up influences provided by separate distractor stimuli, which induces a switch between mental representations driving response selection and thus a stimulus-stimulus conflict (31). It hence seems conceivable that even though former methamphetamine likely present with decreased interference control (as assessed by the stoop task), the ability to guide attention, select a correct response from a subset of alternatives in conflicting situations, and to take previous contextual information into account seem to be relatively preserved.

Yet still, it should not be ignored that the dopaminergic downregulation reported for methamphetamine users seems to partly improve with increasing duration of abstinence. Several studies have demonstrated that the reinstatement of comparatively normal dopamine signaling may take from < 6 months of abstinence to 12 to 17 months of abstinence (20, 56). We therefore conducted add-on analyses of abstinence duration in the methamphetamine consumer’s group only. Because the sample size of n = 12 subjects per group is very small and does likely not allow for valid conclusions due to lack of power, the results should be treated with ample caution and are therefore only presented in the **Supplementary Material**. Due to the relatively large span of abstinence duration in our sample and the rather long abstinence duration of more than two years, our study does not allow for conclusions about the immediate effect of very short abstinence duration (e.g., < 6 months), which may theoretically still be associated with noteworthy response selection deficits.

Lastly, our cross-sectional study design did not allow to examine executive functioning prior to the initiation of methamphetamine use. In contrast to this, a longitudinal design would have allowed for further conclusions on cause and effect. After all, it could also be possible that individuals with low cognitive resilience in form of decreased executive functioning are more likely to either start using drugs like methamphetamines, or maintain their consumption more steadily (18). Another limitation of this study is that even though we took measures to minimize the social desirability bias (please see methods section), applicants may still have answered questions about their substance consumption in a manner that they viewed as favorable with respect to their self-image or the social judgement of others. Moreover, we cannot exclude a selection bias: Prior research in individuals with a history of methamphetamine use has suggested that individuals with the greatest degree of dopamine transporter loss are most likely to not remain abstinent (85), which makes it possible that the subjects in our sample only experienced a comparatively mild degree of dopamine neurotoxicity. Given the heavy and prolonged consumption of approx. 5 years of daily substance abuse in our sample, we however deem it quite unlikely that the participants did not experience any dopamine toxicity at all. While we deem it plausible that methamphetamine-induced changes in control functions rely on modulations in dopaminergic/catecholaminergic signaling, our data does not allow to draw direct conclusions about dopaminergic changes in the investigated patients. Hence, further studies, including molecular imaging approaches, are needed to underpin such claims.

## Conclusion

There is a general consensus that heavy methamphetamine use may cause a broad range of cognitive impairments *via* dopaminergic neurotoxicity, which partly persist during (early) abstinence. In a sample that had been abstinent for an average of 2.7 years, we found that abstinent former methamphetamine users showed no significant impairments in the ability to select a correct response from a subset of alternatives in conflicting situations as well as the ability to take previous contextual information into account, including error processing.

Taken together, our results suggest that former methamphetamine use does not appear to be associated with severe deficits in the ability to shield task-relevant information from distracting input in the self-reporting sample we investigated. In combination with the (previous) finding of worse inhibition/interference control in abstinent consumers, our findings suggest that not all cognitive domains are equally impaired by methamphetamine, possibly because different cognitive processes require different levels of dopamine activity.

## Data Availability Statement

The datasets generated for this study are available on request to the corresponding author.

## Ethics Statement

The studies involving human participants were reviewed and approved by the ethics committee of the TU Dresden, Germany. The patients/participants provided their written informed consent to participate in this study.

## Author Contributions

A-KS, MR, and CB designed and planned the study. A-KS and MR collected the data. A-KS, MR, JE, WB, and AO analyzed and interpreted the data. All authors substantially contributed to the manuscript and approved its publication.

## Funding

This work was partly funded by a grant of the Deutsche Forschungsgemeinschaft (DFG) TRR 265 B07 to A-KS and CB and BE 4045/34-1 to CB.

## Conflict of Interest

The authors declare that the research was conducted in the absence of any commercial or financial relationships that could be construed as a potential conflict of interest.
