# Supplementary material for: Methamphetamine Users Show No Behavioral Deficits in Response Selection After Protracted Abstinence
Source: Front Psychiatry. 2019 Nov 19;10:823. doi: 10.3389/fpsyt.2019.00823 (PMC6877501; doi:10.3389/fpsyt.2019.00823)
Supplement: Supplementary file 1 [file DataSheet_1.pdf]

Supplement

Journal *Frontiers in Addictive Disorders*
Title *Methamphetamine Users Show No Behavioral Deficits in Response Selection After Protracted Abstinence*
Authors *Bensmann W, Ernst J, Rädle M, Opitz A, Beste C, Stock AK*
Affiliation *Cognitive Neurophysiology, Department of Child and Adolescent Psychiatry, Faculty of Medicine, TU Dresden, Dresden, Germany*
Correspondence *Ann-Kathrin.Stock@uniklinikum-dresden.de*

1. Introduction

It should be noted that several studies have shown that the downregulation of the dopamine system improves with protracted abstinence (Volkow et al., 2001; Wang et al., 2004). While these studies have demonstrated remarkable improvement /normalization from < 6 months to 12-17 months of abstinence, it has been reported that residual deficits could still be observed after 12-17 months of abstinence (Volkow et al., 2001; Wang et al., 2004). According to those findings, it can be assumed that neurotoxicity-associated deficits in dopaminergic signaling should be strongest within the first 18 months of abstinence, even though they significantly improve within this period. As there is a lack of comparable data on shorter vs. longer abstinence intervals, we decided to further dissociate the effect of short and long abstinence duration. For this, we split of the sample by abstinence duration and subsequently compared former consumers with no more than 18 months abstinence to those with at least 27 months of abstinence. We hypothesized that individuals with short abstinence duration would differ more strongly from their matched controls than former consumers with long abstinence duration (which might not differ from their matched controls at all).

2. Methods

2.2. Methamphetamine subgroups

Methamphetamine-induced dopaminergic dysfunction has been shown to recover with protracted abstinence, i.e., from < 6 months to 12-17 months (Volkow et al., 2001; Wang et al., 2004). However, comparable data on longer abstinence intervals is still lacking. We therefore decided to use 18 months of abstinence as a cutoff value to split our sample into two (coincidentally) equally large subgroups, i.e. a long and short abstinence group. As none of the included subjects had an abstinence duration between 19 and 26 months, the short abstinence group ended up including all subjects with an abstinence duration of up to 18 months, while the long abstinence group only included individuals with an abstinence duration of at least 27 months.

3. Results

3.1. Sample description of former methamphetamine consumers

Lifetime prevalence of common drug consumption was assessed with the Alcohol, Smoking & Substance Involvement Screening Test (ASSIST). In the methamphetamine consumer group, everyone (n = 24) reported ever having consumed alcohol and tobacco aside from amphetamines and n = 23 also reported having consumed cannabis. Cocaine had been consumed by n = 20, hallucinogens by n = 16, sedatives by n = 10, opiates by n = 9, inhalants by n = 8 and other drugs by n = 3. Aside from these lifetime prevalences, n = 2 reported a risky consumption behavior with regard to tobacco, and n=1 reported risky consumption behavior

with regard to alcohol within the past three months (as defined by a score of at least 27 points). All other participants showed low or medium risk consumption behavior of the above-mentioned drugs in the three months before data acquisition (as defined by the score of at less than 27 points).

Based on the M.I.N.I International Neuropsychiatric Interview, some of the former methamphetamine abusers also showed clinically relevant symptoms qualifying for the following diagnoses: current major depression (*n* = 3), manic episode while consuming methamphetamine (*n* = 5), hypomanic episode while consuming methamphetamine (n=2), current psychotic episode (*n* = 4), psychotic episode while consuming methamphetamine (*n* = 9), and antisocial personality disorder (*n* = 8). Yet, these diagnoses need to be treated with ample caution as the M.I.N.I is a short screening and cannot capture all background information and symptoms required for a full, validated diagnosis. Also, criminal behavior associated with drug seeking or obtaining money for the drug may overlap with the symptoms of antisocial personality disorder, especially when consumption is started at a reasonably young age.

Furthermore, the methamphetamine subgroups differed significantly from each other with respect to abstinence duration [$t(22)= -4.757$; $p < .001$]: The short abstinence group was abstinent for 10.5 months (± 5.9; range 1.5 to 18) and the long abstinence group was abstinent for 53.3 months (± 30.6; range 27 to 120). All other assessed methamphetamine-related information as well as age and sex did not differ between the short and long abstinence groups (please see Table S1).

**Measure**	**Entire METH group**	**METH, short abstinence only**	**METH, long abstinence only**	**Subgroup differences (short vs. long)**
**General Measures**				
Sex	n = 13 ♂<br>n = 11 ♀	n = 7 ♂<br>n = 5 ♀	n = 6 ♂<br>n = 6 ♀	p = .698
Age	29.5 ± 5.0	29.3 ± 4.8	29.7 ± 5.4	p = .845
Age at first consumption (years)	18.4 ± 3.9	18.6 ± 3.7	18.2 ± 4.1	p = .781
Duration of consumption (months)	86.2 ± 47.8	97.9± 39.9	74.5 ± 53.7	p = .239
Abstinence duration (months)	31.9 ± 30.6	10.5 ± 5.9	53.3 ± 30.5	**p < .001***
Estimated total amount of amphetamines consumed (gramm)	1882.0 ± 3930.2	2206.0± 5127.6	1585.0 ± 2605.6	p = .714
**Measures assessed for self-declared time period of non-addicted recreational use (as defined by the absence of craving, subjective withdrawal symptoms, adverse social or occupational consequences and drug-related crime)**				
Average pause between consumption incidents (hours)	220.5 ± 234.2	252.0 ± 309.0	192.0 ± 148.0	p = .571
Average amount of methamphetamine consumed in a single consumption incident (gramm)	0.344 ± 0.270	0.318 ± 0.187	0.370± 0.341	p = .663
Maximal amount of methamphetamine consumed in a single day (grams)	1.004 ± 1.034	0.722 ± 0.493	1.286 ± 1.351	p = .407
**Measures assessed for self-declared time period of "addicted" use (as defined by craving, subjective withdrawal symptoms, and adverse social or occupational consequences)**				
Duration of subjective methamphetamine addiction & consumption (months)	59.0 ± 36.1	70.3 ± 41.2	48.6 ± 28.7	p = .155
Average pause between consumption incidents (hours)	61.0 ± 74.3	80.0 ± 90.9	42.0 ± 49.8	p = .217
Average amount of methamphetamine consumed in a single consumption incident (gramm)	0.548 ± 0.602	0.637 ± 0.662	0.459 ± 0.549	p = .482
Maximal amount of methamphetamine consumed in a single day (grams)	1.345 ± 0.756	1.412 ± 0.793	1.279 ± 0.746	p = .676
Average number of consumption incidents on days where methamphetamine was consumed	6.02 ± 2.76	6.62 ± 3.14	5.36 ± 2.23	p = .284

Table S1: Details on methamphetamine abuse in the abstinent consumer group. Mean ± standard deviation are provided for all metric variables, absolute numbers are provided for sex. A χ^2 test of independence was used to investigate sex differences between the two abstinence groups. Mann-Whitney-U-tests were used to investigate differences between the two abstinence groups in all other measures. With respect to the consumed amounts, it should be noted that the methamphetamine illegally sold in Saxony is deemed to be quite pure and rarely found to be strongly diluted. It should furthermore be considered that this information was collected retrospectively and based on the consumers' subjective memory, which may deviate from the actual amounts and consumption frequencies.

3.2 Questionnaires and neuropsychological tests

We found that former consumers exhibited worse inhibitory control, as assessed with the Stroop test. In the conflict condition (i.e., in case of a mismatch between word meaning and font color), the meth group showed significantly slower task performance (34.55 ms ± 1.21) than the control group (29.23 ms ± 1.21) ($t(46) = -3.05$, $p = .004$). They also had significantly higher BDI scores (3.98; ± 4.35; range 0 to 18) than the control group (8.29; ± 5.06; range 2 to 21) ($t(46) = -3.16$, $p = .003$). We did however choose not to correct for this, as anhedonia and depressive symptoms are likely to be rooted in the very same monoaminergic deficits that we aimed to investigate in our study. Additional correlational analyses conducted only in the meth group showed that neither age of first methamphetamine consumption, nor the duration of consumption (either total time or limited to the time of self-reported addiction), nor abstinence duration, were significantly correlated with the Stroop or BDI measures (all p ≥ 187). We found no significant group differences between former methamphetamine consumers in the short and long abstinence group with any neuropsychological test or questionnaires (all p ± .243). All neuropsychologic test results are provided in Tabel S2.

Test score	Control group (n = 24)	Meth group (n = 24)	p value	Cohen's d
TMT (B minus A) / sec	26.67 ± 2.90	26.93 ± 2.84	.950	-0.018
Stroop conflict / sec	29.23 ± 1.05	34.55 ± 1.38	**.004 ***	-0.883
Digit span (max. forward span / items)	6.50 ± 0.15	6.21 ± 0.22	.296	0.304
Digit span (max. backward span / items)	4.96 ± 0.23	4.67 ± 0.29	.443	0.222
Corsi block span (max. forward span / items)	6.00 ± 0.20	5.88 ± 0.19	.655	0.125
Corsi block span (max. backward span / items)	5.96 ± 0.26	5.58 ± 0.24	.301	0.306
d2 F% score	9.69 ± 1.41	11.72 ± 2.40	.470	-0.214
BDI score	3.98 ± 0.88	8.29 ± 1.03	**.003 ***	-0.914

Table S2: Neuropsychologic tests and depression. Independent comparisons showed no group differences for the difference between response times in task version B and A of the Trail Making Test (TMT), the percentage of overall errors in the d2 test (F%), or the maximum number of correctly recalled items in the digit span and Corsi block span tests. Yet, the abstinent methamphetamine consumer group showed significantly slower task performance in the conflict condition of the verbal Stroop task as well as significantly higher scores in the Beck's Depression Inventory (BDI), as compared to the matched controls.

3.3 Add-on analyses: Abstinence duration in methamphetamine consumers

The short abstinence subgroup used for add-on analyses was abstinent for 10.5 months (± 5.9; range 1.5 to 18) and the long abstinence subgroup was abstinent for 53.3 months (± 30.6; range 27 to 120).

In the add-on analysis of the effects of abstinence duration on the behavioral data obtained in the flanker task (i.e., when excluding all controls), no main effects or interactions of the abstinence subgroup factor were significant for accuracy (all F ≤ 3.287; p ≥ .083), hit RTs (F ≤ 2.052; p ≥ .166), and post-error slowing (PES) (F ≤ 0.874; p ≥ .360). The results obtained in

our Bayesian analysis of abstinence subgroup effects (as suggested by Masson, 2011) are summarized in Table S3.

	Accuracy in %	*RTs in ms*	*PES in ms*
Main effect abstinence subgroup	pBIC(H0|D) = .659	pBIC(H0|D) = .809	pBIC(H0|D) = .824
Current trial x abstinence subgroup	**pBIC(H0|D) = .113**	pBIC(H0|D) = .857	pBIC(H0|D) = .753
Previous trial x abstinence subgroup	**pBIC(H0|D) = .053**	pBIC(H0|D) = .628	
Previous trial x current trial x abstinence subgroup	pBIC(H0|D) = .869	pBIC(H0|D) = .255	

Table S3: Bayesian analyses for all effects involving the abstinence subgroup factor in former methamphetamine consumers. Please note that no data on the effects of previous trial are reported for post-error slowing (PES), as we did not have enough incorrect responses to reliably analyze this factor.

While most of these results provide greater evidence for the null hypothesis (i.e. no differences between abstinence subgroups) and thus the rejection of the alternative hypotheses (i.e. differences between abstinence subgroups), we obtained positive evidence for an interaction of previous trial x abstinence subgroup as well as an interaction of current trial x abstinence subgroup. As these factors did not reach significance in our prior ANOVA analyses, we refrained from calculating post-hoc tests. On the descriptive level (see Figure S1), it however seems that consumers with short abstinence duration tended to perform worse (i.e., less accurately) than consumers with long abstinence duration whenever at least one kind of conflict was present (i.e., in the iC, cI, and iI conditions), but less so in the absence of conflicts (i.e., the cC condition). Also, the largest abstinence subgroup difference by absolute numbers can be seen when both kinds of conflict are combined (i.e., in the iI condition).

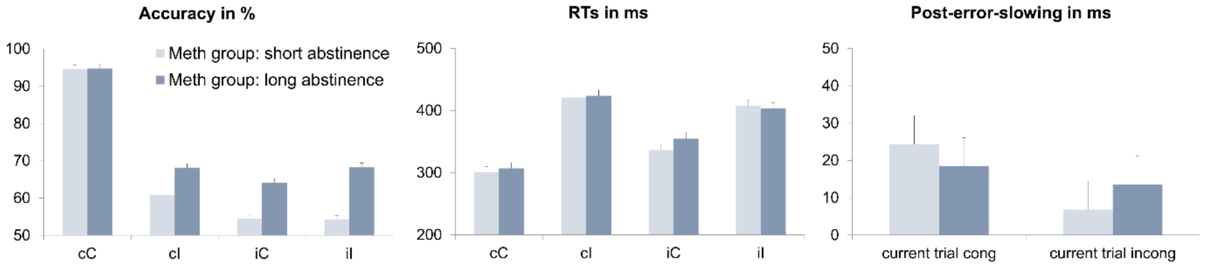

Figure S1
Behavioral results. In our add- on analyses of the consumer group only, abstinence subgroup (short vs. long abstinence duration) did not significantly interact with any of the other factors. Bayesian analyses however suggested that an interaction of abstinence subgroup x current trial, as well as an interaction of abstinence subgroup x previous trial were more likely than the null hypothesis in the accuracy measure (left graph). Error bars show the standard error of the mean (SEM) as a measure of variability.

4. Discussion

The dopaminergic system has been demonstrated to substantially improve/normalize with protracted abstinence. Therefore, we dissociated the effect of short and long abstinence duration by means of a sample split. Here, we had hypothesized that former abstinent methamphetamine

users should show relatively larger flanker and Gratton effects after short abstinence, as compared to long abstinence.
Like the analyses of the consumption group factor, our add-on analyses of abstinence duration did not reach significance. This was however only partly confirmed by Bayesian analyses, where we obtained positive evidence for the alternative hypothesis being true for the interaction of abstinence duration and current trials, as well as for the interaction of abstinence duration and previous trial. As these factors had failed to reach significance in our prior ANOVA analyses, we decided to refrain from calculating post-hoc tests. On the descriptive level (see Fig. S1), it however seemed that consumers with short abstinence duration tended to perform worse than consumers with long abstinence duration whenever at least one kind of conflict was present, but not necessarily in the absence of current trial and previous trial conflicts. As a consequence, both the flanker effect and the Gratton effect appeared to be slightly larger in the short abstinence group than in the long abstinence group. Given that these differences failed to reach significance in parametric testing, they should however be treated with ample caution. As each of the abstinence subsamples only contained n=12 subjects, we additionally determined whether our sample had been sufficiently powered using post-hoc power analyses employing G-power software (http://www.gpower.hhu.de/) (Faul et al., 2009). These analyses informed us that the sample was most likely sufficiently powered (both $1-\beta > 90\%$ at $\alpha = 5\%$ when entering the obtained effect sizes [current trial: f = 0.35; previous trial: 0.39] and within-subject inter-correlations [current trial: r = 0.506; previous trial: 0.928]). Yet still, it should be noted that this sample might still be too small to allow for valid statements about the reported effect. Hence, further studies with larger samples of short and long abstinence duration will be needed to see whether this claim can be substantiated (Brysbaert, 2019). Taken together, these findings suggest that even though a significant difference between short and long abstinence might have been observed in a comparable larger sample, this effect is most likely not very large/meaningful. In this context, it should however be noted that the average abstinence duration of our study sample was 2.7 years and therefore well beyond the time marks that usually show the greatest improvements in dopaminergic functioning (Volkow et al., 2001; Wang et al., 2004). While our add-on analyses accounted for abstinence duration, the short abstinence subgroup had already been abstinent for an average of 10.5 months. It is therefore possible that the dopaminergic system had already partly recovered after this time of abstinence to the point where the abstinence subgroup differences would no longer reach significance. In line with this assumption, Bayesian analyses still suggested some behavioral impairments in the face of response conflicts. Hence, our study does not allow for conclusions about the immediate effect of very short abstinence duration (e.g., < 6 months), which may theoretically still be associated with response selection deficits.

5. Conclusion

Add-on analyses of abstinence duration found no significant performance differences between consumers with short and long abstinence durations (average of 10.5 vs. 53.3 months). Bayesian analyses still suggested that individuals who have been abstinent for less than 10 months might show slight impairments response selection, given that methamphetamine-induced dopaminergic toxicity tends to improve with abstinence duration. Yet, these findings need to be investigated in a much larger sample to allow for valid conclusions.

References

Brysbaert, M. (2019). How many participants do we have to include in properly powered experiments? A tutorial of power analysis with reference tables. *J. Cogn.* 2, 16. doi:10.5334/joc.72.

Faul, F., Erdfelder, E., Buchner, A., and Lang, A.-G. (2009). Statistical power analyses using G*Power 3.1: tests for correlation and regression analyses. *Behav. Res. Methods* 41, 1149–1160. doi:10.3758/BRM.41.4.1149.

Masson, M. E. J. (2011). A tutorial on a practical Bayesian alternative to null-hypothesis significance testing. *Behav. Res. Methods* 43, 679–690. doi:10.3758/s13428-010-0049-5.

Volkow, N. D., Chang, L., Wang, G.-J., Fowler, J. S., Franceschi, D., Sedler, M., et al. (2001). Loss of Dopamine Transporters in Methamphetamine Abusers Recovers with Protracted Abstinence. *J. Neurosci.* 21, 9414–9418. doi:10.1523/JNEUROSCI.21-23-09414.2001.

Wang, G.-J., Volkow, N. D., Chang, L., Miller, E., Sedler, M., Hitzemann, R., et al. (2004). Partial Recovery of Brain Metabolism in Methamphetamine Abusers After Protracted Abstinence. *Am. J. Psychiatry* 161, 242–248. doi:10.1176/appi.ajp.161.2.242.
